# Do Different Durations of Hyperbaric Oxygen Therapy Affect the Microleakage of Bulk-Fill Composites?

**DOI:** 10.3390/jfb17050209

**Published:** 2026-05-01

**Authors:** Cemile Kedici Alp, Sena Sumra Kaçmaz, Ayşenur Yazım, Zeynep Aydin, Recep Özkan

**Affiliations:** 1 Department of Restorative Dentistry, Faculty of Dentistry, Gazi University, Emek, Ankara 06490, Türkiye; senasumra@gazi.edu.tr (S.S.K.); aysenuryazim@gazi.edu.tr (A.Y.); zeyneporan@gazi.edu.tr (Z.A.); 2 Department of Underwater and Hyperbaric Medicine, Erzurum City Hospital, Erzurum 25240, Türkiye; recep.ozkan4@saglik.gov.tr

**Keywords:** bulk-fill, hyperbaric oxygen therapy, microleakage, resin composites

## Abstract

This in vitro study evaluated the effect of exposure duration (5, 20, and 40 days) to constant increased ambient pressure (2.4 atmospheres absolute; ATA) on microleakage at the dentin–composite interface of teeth restored with two bulk-fill composites. Specimens stored in distilled water at atmospheric pressure (1 atm) served as controls. A total of 192 extracted human molars with standardized Class V cavities were randomly assigned to two groups: sonic-activated bulk-fill composite (SonicFill) or conventional bulk-fill composite (Filtek One Bulk Fill). Each group was subdivided into controls maintained under atmospheric pressure (1 atm) and specimens under hyperbaric pressure (2.4 ATA), and exposed for 5, 20, or 40 days (total of 12 groups, *n* = 16 per group). Microleakage was assessed using the dye penetration method and scored under a stereomicroscope according to ISO criteria. Statistical analyses were performed using Fisher’s Exact chi-squared and Fisher–Freeman–Halton Exact tests (α = 0.05). No significant differences were found between materials or pressure conditions at 5 and 20 days (*p* > 0.05). After 40 days, both composites showed significantly higher microleakage at increased pressure than atmospheric controls (*p* < 0.05). Microleakage increased over time in the hyperbaric groups, while no time-dependent changes occurred at atmospheric pressure. After 40 days, prolonged exposure to elevated pressure increased microleakage, whereas shorter exposure produced no significant changes. Both materials demonstrated similar susceptibility to pressure-related deterioration.

## 1. Introduction

Microleakage stands out as a critical parameter to evaluate the physical properties of restorative materials [1]. The incremental placement technique for resin composites has been recommended for several years to prevent this effect. This technique helps distribute stress evenly and enhances the adaptation of composite resin to the tooth surface, prolonging the longevity of the restoration. However, layering increases clinical procedure time and can lead to interlayer contamination, adhesive failure, and manipulation difficulties within narrow cavities [2]. To reduce the impact of these disadvantages, bulk-fill composites have recently been developed [3,4]. Bulk-fill composites are designed to simplify restorative procedures, as they can be applied in a single 4–5 mm layer in posterior cavities [5,6].

The use of such bulk-fill composite resins has increased in recent years. Development has focused on improving the physical, chemical, and mechanical properties of these materials, leading researchers to investigate different application techniques. Sonic activation is one of the methods developed to reduce the viscosity of composites. Sonic activation can speed up the restoration process and help the material adapt to the cavity walls [7,8]. SonicFill differs from other bulk-fill materials in its application method, and 5 mm can be applied in a single layer. The manufacturer asserts that the specialized handpiece used for this technique decreases the material’s viscosity by up to 87%, and the increased flowability enhances adaptation to the cavity walls, thereby helping to reduce microleakage [9,10].

Barodontalgia refers to tooth pain that occurs as a result of variations in ambient pressure. In 1965, Shiller examined dental pain in hyperbaric settings and concluded that comparable mechanisms cause toothache in hypobaric and hyperbaric conditions. This led to the use of terminology in which ‘baro’ indicates changes in pressure [11,12]. Alterations in atmospheric pressure lead to changes in gas volume within closed, rigid body cavities. These variations may give rise to several side effects [13]. Scuba divers, patients undergoing hyperbaric oxygen therapy (HBOT), and miners are among the groups that may experience barotrauma [14].

HBOT is widely applied in various medical practices, including dental, reconstructive, and wound-healing procedures. However, protocols are not standardized, and parameters such as session duration, pressure, and number of treatments vary depending on the condition being treated [15]. For example, clinical studies have reported session durations ranging from 5 to 40 days, with the number of treatments adapted to individual patient needs [16]. Here, 5-, 20-, and 40-day HBOT exposure periods were selected to represent early, intermediate, and prolonged cumulative hyperbaric exposure, rather than to reproduce a single disease-specific treatment regimen. HBOT protocols vary considerably across accepted indications, depending on the underlying condition and treatment goals. According to the Undersea and Hyperbaric Medical Society indications resource, HBOT can be used for a broad range of acute and chronic conditions, with treatment protocols delivered at pressures generally ranging from 1.9 to 3.0 atmospheres absolute (ATA) [17]. For wound-related and other chronic indications, HBOT is commonly administered as daily sessions over 20 to 40 consecutive treatments [18]. For chronic refractory osteomyelitis, treatment courses may extend to 40 to 60 sessions [19]. Therefore, 5 days was chosen to reflect an early cumulative exposure phase, 20 days an intermediate exposure stage, and 40 days a prolonged exposure period comparable to longer clinical treatment courses. This design allowed us to assess whether microleakage changes progressively with increasing cumulative hyperbaric exposure.

Differences in HBOT protocols may alter systemic and dental responses and related effects. Several adverse effects have been identified related to the application of HBOT. These include middle ear barotrauma, sinus and paranasal sinus barotrauma, ocular side effects, hypoglycemia, oxygen-induced seizures, and claustrophobia [15]. Dental effects mainly relate to restorations, especially composite restorations and dental implants. In the literature, microleakage of dental composites has been studied under scuba diving conditions, with increased microleakage reported in teeth exposed to 4 bar pressure [20]. These findings indicate that microleakage in different composite materials may be affected by pressure levels and exposure duration.

Mechanistic theories may explain why hyperbaric conditions affect the resin–dentin interface. Polymerization shrinkage can generate contraction stress and produce interfacial gaps in bonded restorations; reduced gap formation and better internal adaptation are reportedly closely related to lower polymerization shrinkage stress and strain [21,22]. Under hyperbaric conditions, pre-existing voids or interfacial defects may therefore become more critical. In support of this interpretation, simulated hyperbaric conditions in vitro resulted in greater dye percolation and loss of sealing at the dentin–composite interface, particularly in porous restorations [23]. These findings suggest that structural defects and inadequate marginal adaptation may increase the susceptibility of the adhesive interface to hyperbaric stress.

Although the mechanical properties, polymerization shrinkage, and microleakage of bulk-fill composites have been previously studied, the impact of hyperbaric conditions on the microleakage of SonicFill composites has not been reported in the literature [23,24]. The present study was designed to assess the effect of pressure increases on microleakage occurring at the dentin–composite resin interface of teeth restored with two different bulk-fill resin composites. The null hypotheses of the present study were:

1. Different durations of hyperbaric oxygen exposure have no effect on microleakage.
2. Different bulk-fill composite resins used under hyperbaric oxygen exposure have no effect on microleakage.

## 2. Materials and Methods

### 2.1. Sample Selection

The study received ethical approval from the Ethics Committee of Gazi University Faculty of Dentistry (4 August 2025-E.1300584). Power analysis assuming 80% power (power = 0.80), a 5% significance level (α = 0.05), and an effect size (d) for microleakage levels between groups of 0.4 indicated that a minimum of n = 16 specimens per subgroup was required. Sample size calculations were performed using the G*Power 3.1.9.4 software package (Franz Faul, Universität Kiel, Kiel, Germany). This study included 192 non-carious maxillary and mandibular molar teeth that had been extracted within the last six months for orthodontic or periodontal reasons, all obtained from adult patients. Following the removal of all residual soft tissues and polishing with pumice, the samples were kept in distilled water until their use in the experimental procedures.

### 2.2. Cavity Preparation

For each tooth, a standardized Class V cavity was prepared on the dentine surface 1 mm coronally from the cementoenamel junction, with dimensions of 4 mm (mesiodistally), 3 mm (occluso-gingivally), and 2 mm in depth, based on previous studies. All measurements were verified using a periodontal probe. All cavity preparations were performed by a single calibrated operator [25,26]. The cavity preparations were performed under water cooling using round diamond and fissure burs. Following cavity preparation, a 0.5 ± 1 mm bevel was applied to the enamel margins using a yellow-banded flame-shaped bur. The bur was replaced after every five cavities.

### 2.3. Restorative Procedure

Class V cavity dimensions were measured using a digital caliper (Insize 1112-150, Insize Inc., Palo Alto, CA, USA) and depths using a periodontal probe. The 192 prepared teeth were randomly divided into 12 experimental groups (n = 16 per group). All cavities were prepared by a single operator, and the restorations were completed by a single operator, to ensure standardization. A universal adhesive (G2 Bond, GC Corp., Tokyo, Japan, Lot No. 2210071) was applied to all specimens using the selective-etch technique with 37% phosphoric acid. It was then light-cured, following the manufacturer’s instructions, for 10 s using a D-Light Pro (D-Light Pro LED, GC Europe, Leuven, Belgium) curing unit with an irradiance of approximately 1200 mW/cm^2^. In total, 96 teeth were restored with sonic-activated composite (SonicFill, Kerr Corp., Orange, CA, USA, Lot No. 9591358) and 96 teeth with bulk-fill composite (Filtek Bulk Fill, 3M ESPE, St. Paul, MN, USA, Lot No. 10639667) using a D-Light Pro, following the manufacturer’s instructions. The composite resins were light-cured for 20 s. After the restorations were performed, all samples were kept in distilled water for 24 h to allow post-polymerization.

### 2.4. Experimental Group Distributions

**Figure 1:**
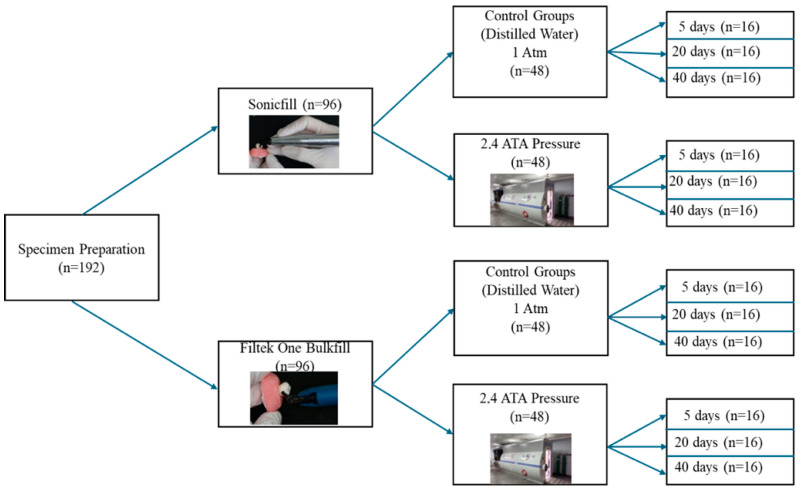
Experimental design of the study.

The main groups (sonic-activated and bulk-fill) were each further divided into six subgroups. For each resin, teeth in three control subgroups were kept in distilled water at atmospheric pressure (1 atm) for 5, 20, and 40 days, respectively. The remaining three subgroups were exposed to hyperbaric conditions (2.4 ATA) for 5, 20, and 40 days, respectively. These intervals were selected to represent short-, intermediate-, and prolonged cumulative HBOT exposure periods reported in clinical practice. The hyperbaric chamber was pressurized using ambient air (approximately 21% oxygen and 79% nitrogen), and the exposure was not continuous. The protocol consisted of a 15 min compression phase to reach the target pressure, followed by 90 min at the treatment depth (2.4 ATA), before a 15 min decompression phase, for a total session duration of 120 min. This procedure was applied for each session. The experimental design of the study is presented in Figure 1.

### 2.5. Dye Penetration

Following the full experimental protocol, the external surface of each sample was covered with two layers of nail varnish, leaving a 1 mm-wide margin around the restoration interface uncoated. The specimens were then stored in a 0.5% basic fuchsin dye solution in an incubator at 37 °C. Following the dye procedure, the specimens were rinsed with water and embedded in a cold-curing acrylic resin. They were sectioned with a diamond disc along the mesio-distal axis, aligned with the center of the restoration. Microleakage was evaluated under a stereomicroscope at 20× magnification by an examiner who was blinded to group allocation, using predefined scoring criteria at standardized occlusal and gingival locations. To assess reproducibility, the same blinded examiner repeated the scoring procedure on a second occasion, and intra-examiner agreement was calculated using Cohen’s kappa coefficient (κ = 0.901, SE = 0.033, *p* < 0.001). Of the two sections obtained from each tooth, one was selected for evaluation, and the higher score between gingival and occlusal microleakage was taken as the reference value.

Dye penetration at the gingival wall was evaluated and recorded according to the ISO scoring system (ISO/TS 11405:2003) [27]:

Score 0 = no dye penetration;

Score 1 = dye penetration up to half of the gingival wall;

Score 2 = dye penetration extending beyond half of the gingival wall but not reaching the axial wall;

Score 3 = dye penetration reaching both the cervical and axial walls. These results were then determined and documented as the microleakage outcomes of the cavities.

### 2.6. Statistical Analysis

The study findings were analyzed using IBM SPSS Statistics 27 software. Prior to the main statistical analysis, intra-examiner reliability for microleakage scoring was assessed using Cohen’s kappa. Descriptive statistics for each group were reported as median and interquartile range (IQR), given the ordinal nature of the outcome measure. Score distributions were summarized as frequencies and percentages. Because microleakage scores constitute an ordinal scale and the distribution of higher score categories was sparse across groups, distribution-free rank-based tests were employed throughout. For pairwise comparisons between two independent groups, the Mann–Whitney U test was used. For comparisons across three time points within the same composite and application condition, the Kruskal–Wallis test was applied, followed by Dunn–Bonferroni post hoc tests in cases of a significant omnibus result. Effect sizes were reported as the rank-biserial correlation coefficient (r) for Mann–Whitney U comparisons, calculated as r = 1 − 2U/(n_1_·n_2_), and as eta-squared (η^2^) for Kruskal–Wallis analyses, calculated as η^2^ = (H − k + 1)/(n − k). To account for multiple comparisons, the Bonferroni correction was applied separately within each comparison family, as each table addresses a distinct research question. A corrected threshold of *p* < 0.0083 was used for the six pairwise Mann–Whitney comparisons in each of Table 1 and Table 2, and a threshold of *p* < 0.0125 for the four omnibus Kruskal–Wallis tests in Table 3. Results were otherwise considered statistically significant at *p* < 0.05.

## 3. Results

Intra-examiner reliability for microleakage scoring was assessed prior to analysis by having the same examiner re-evaluate all sections on a second occasion. Cohen’s kappa was 0.901 (SE = 0.033, *p* < 0.001), indicating excellent reproducibility.

Table 1 presents the comparison of microleakage scores between SonicFill and Filtek One Bulk Fill, according to application type and time interval. No statistically significant differences were observed between the two composite groups at any time point under pressure or distilled water conditions (all *p* > 0.05, after Bonferroni correction). Effect sizes were negligible to small across all comparisons (r = 0.062–0.262). The largest effect was observed at Day 40 under pressure (r = 0.262), but the difference was not significant. These findings support the second null hypothesis, indicating that material type did not influence microleakage outcomes.

**Figure 2:**
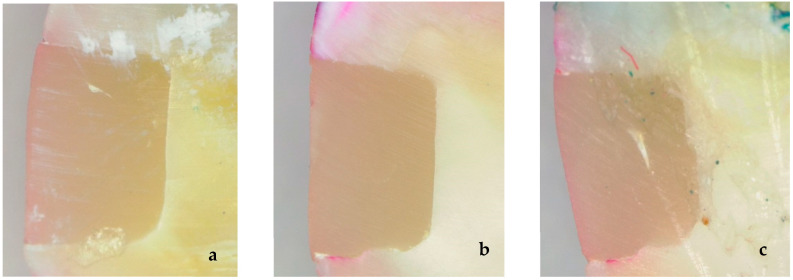
(**a**) Score 0—No evidence of microleakage, (**b**) Score 1—Microleakage involving less than one-half of the cavity wall, (**c**) Score 2—Microleakage involving more than one-half of the cavity wall.

As shown in Table 1, in the groups exposed to 2.4 ATA pressure for 5 days, the SonicFill composite had 14 specimens with a score of 0, and two specimens with a score of 1. Meanwhile, the Filtek One composite had 13 specimens with a score of 0, and three specimens with a score of 1. At 20 days of exposure to 2.4 ATA pressure, 12 SonicFill specimens had a score of 0, four specimens had a score of 1, and no specimens had a score of 2. In comparison, Filtek One presented 11 specimens with a score of 0, four specimens with a score of 1, and one specimen with a score of 2. Following 40 days of 2.4 ATA pressure exposure, the SonicFill group exhibited seven specimens with a score of 0, three specimens with a score of 1, and six specimens with a score of 2. The Filtek One group contained three specimens with a score of 0, four specimens with a score of 1, and nine specimens with a score of 2. In contrast, in the distilled water (control) groups, the 5-day results with SonicFill showed 16 specimens with a score of 0 and no specimens with higher scores; meanwhile, the Filtek One group had 15 specimens with a score of 0 and one specimen with a score of 1. At 20 days, the SonicFill group contained 14 specimens with a score of 0 and two specimens with a score of 1, while Filtek One had 13 specimens with a score of 0 and three specimens with a score of 1. At 40 days, SonicFill had 13 specimens with a score of 0 and three specimens with a score of 1; Filtek One had 12 specimens with a score of 0 and four specimens with a score of 1. The microleakage levels are presented in Figure 2.

Table 2 shows the comparison of microleakage scores between the pressure (2.4 ATA) and distilled water control (1 Atm) conditions for each composite at each time point. For SonicFill, no statistically significant differences were detected between the pressure and control conditions at Day 5 or Day 20 (*p* = 0.564 for both; r = 0.125). At Day 40, the proportion of Score 2 observations in the pressure group (37.5%) was markedly higher than in the control group (0%), and a medium-to-large effect was observed (r = 0.445); however, this difference was not significant after Bonferroni correction (*p* = 0.032; corrected threshold *p* < 0.0083).

For Filtek One Bulk Fill, no significant differences were found between conditions at Day 5 (*p* = 0.564, r = 0.125) or Day 20 (*p* = 0.515, r = 0.137). At Day 40, a statistically significant and large effect was observed (*p* < 0.001, r = 0.703), which remained significant after Bonferroni correction. The proportion of Score 2 in the pressure group (56.2%) was substantially higher compared to that of the control group (0%), and the median microleakage score increased from 0 (IQR: 0–1) in the control group to 2 (IQR: 1–2) under pressure. These results indicate that prolonged hyperbaric exposure significantly compromised marginal sealing in Filtek One Bulk Fill, leading to rejection of the first null hypothesis for this material at the 40-day time point.

Table 3 summarizes the time-course of microleakage scores within each composite and application group. For SonicFill under pressure (2.4 ATA), the Kruskal–Wallis test revealed a statistically significant difference over time (H = 9.744, *p* = 0.008, η^2^ = 0.172), indicating a medium-to-large effect of exposure duration. Dunn–Bonferroni post hoc tests identified a significant difference between Day 5 and Day 40 (*p* = 0.009), while the Day 5 vs. Day 20 (*p* > 0.999) and Day 20 vs. Day 40 (*p* = 0.061) comparisons were not significant after correction. Score 2 was absent at Day 5 and Day 20, but was observed in 37.5% of specimens at Day 40, with the median score increasing from 0 (IQR: 0–0) at Day 5 to 1 (IQR: 0–2) at Day 40. No significant time-dependent change was observed in the SonicFill distilled water control group (H = 3.060, *p* = 0.216, η^2^ = 0.024).

For Filtek One Bulk Fill under pressure, the Kruskal–Wallis test yielded a significant result (H = 18.437, *p* < 0.001, η^2^ = 0.365), corresponding to a large effect size. Post hoc comparisons confirmed significant differences between Day 5 and Day 40 (*p* < 0.001) and between Day 20 and Day 40 (*p* = 0.002); the Day 5 vs. Day 20 comparison was non-significant (*p* > 0.999). Score 2 was absent at Day 5, observed in 6.2% of specimens at Day 20, and increased markedly to 56.2% at Day 40, with the median score rising from 0 (IQR: 0–0) at Day 5 to 2 (IQR: 1–2) at Day 40. No significant time-dependent change was observed in the Filtek One Bulk Fill distilled water control group (H = 2.056, *p* = 0.358, η^2^ = 0.001). Across both materials, microleakage increased progressively with exposure duration under hyperbaric conditions, whereas control group scores remained stable throughout the observation period, providing strong internal validity for the pressure effect.

## 4. Discussion

HBOT is used to treat a variety of medical conditions. However, there is no single standard protocol for its application. The duration and method of HBOT may change depending on the condition treated, with periods generally ranging from 5 to 40 days. These differences may influence the effects of HBOT on the body and specifically dental tissues [28]. Here, 5-day exposure was used to represent treatment for acute conditions, such as carbon monoxide poisoning and central retinal artery occlusion, for which HBOT is applied for a short period [29]. For these indications, treatment usually involves only a few sessions. The 20-day exposure was designed to represent a moderate treatment duration, such as that used for sudden sensorineural hearing loss [30]. The 40-day exposure was designed to represent long-term HBOT indications requiring repeated sessions, such as diabetic foot ulcers, radiation necrosis, and chronic refractory wounds [31]. These experimental exposures were selected to reflect short-, intermediate-, and prolonged cumulative HBOT exposure periods reported in clinical practice, rather than to replicate a single disease-specific schedule. This approach allowed for evaluation of whether cumulative hyperbaric exposure progressively affects the restoration–tooth interface over time [16,29,32]. No significant increase in microleakage was observed after 5 or 20 days of exposure. However, a significant increase was detected in both composite materials after 40 days. These findings indicate that the effect of hyperbaric pressure on the restoration–tooth interface is progressive and time-dependent. These findings align with the literature describing HBOT as a repetitive and long-term treatment. Careful follow-up and appropriate material selection may therefore be important for maintaining the marginal integrity of dental restorations in patients who may undergo frequent or long-term HBOT [33,34].

In this study, microleakage was evaluated at 5, 20, and 40 days to determine how two bulk-fill composites respond to prolonged pressure. No significant changes were observed at the early time points, but a noticeable increase in microleakage appeared after 40 days of hyperbaric exposure. In the SonicFill-applied groups, the microleakage score of 2 on day 40 (37.5%) was considerably higher than that on day 5 (0%) and day 20 (0%). In contrast, no significant changes were observed over time when these specimens were immersed in distilled water. Similarly, for Filtek One Bulk-Fill composite, microleakage scores of 2 on day 40 (56.2%) were considerably more common than on days 5 (0%) and 20 (6.3%). Again, no significant differences were observed over time when teeth treated with the composite were immersed in distilled water (*p* > 0.05). These data led to the rejection of the first null hypothesis, indicating that pressure has a progressive effect on the microleakage of these composites. This delayed degradation is consistent with the fact that repeated compression and decompression can slowly weaken the adhesive interface over time [20,35].

In microleakage studies, dye penetration results can be assessed using a scoring system—measuring the percentage of dye infiltration—or with spectrophotometric analysis [36]. The ISO/TS 11405:2003 standard, used as the scoring method here, provides an objective system for microleakage assessment using dye penetration tests, allowing results from different studies to be reliably compared. The standard also defines recommended test methods for evaluating the adhesive bond between restorative materials and tooth structures, including enamel and dentin, which facilitates the use of consistent methodologies across research [27].

Even small gaps that form during composite placement can expand with changes in gas pressure, subsequently weakening the seal at the margins [24]. This mechanism is consistent with previous reports describing barotrauma-associated restoration failures; trapped air expands and contracts during environmental pressure changes, according to Boyle’s law, ultimately weakening the tooth–restoration interface [23,24,37]. These findings confirm the idea that exposure duration is a critical determinant of adhesive stability under hyperbaric conditions. Polymerization shrinkage stress should also be considered when interpreting the present findings. During curing, volumetric contraction occurs simultaneously with an increase in elastic modulus, generating stress at the bonded tooth–restoration interface. When this stress approaches or exceeds bond strength, interfacial debonding and marginal gap formation can occur, creating pathways for subsequent microleakage [38,39]. Previous work has further shown that composites with greater polymerization shrinkage stress exhibit more tooth–composite interfacial debonding [39]. In the present study, the slightly lower early microleakage values observed for SonicFill may be related to improved initial adaptation and lower polymerization shrinkage behavior [40]; meanwhile, Filtek One Bulk Fill incorporates stress-relieving monomer technology intended to reduce shrinkage stress [41]. However, after 40 days of hyperbaric exposure, both materials exhibited increased microleakage. This suggests that polymerization shrinkage stress may have contributed to the formation of initial interfacial defects, while repeated hyperbaric pressure exposure progressively exacerbated these defects over time. Therefore, the observed leakage pattern is most likely explained by the combined effect of polymerization-related interfacial stress and long-term pressure-induced fatigue, rather than by either mechanism alone [20,22,39].

The present findings are unlikely to be explained by viscosity alone. Marginal integrity is likely determined by the combined influence of placement viscosity and initial cavity adaptation, filler loading, polymerization shrinkage behavior, and resin-matrix chemistry. Sonic activation temporarily reduces the viscosity of SonicFill during placement, which may improve flow and initial cavity adaptation [9,10]. In addition, the relatively low polymerization shrinkage reported for SonicFill may be related, in part, to its high filler content [40]. Filtek One Bulk Fill, in contrast, was developed with stress-relieving monomer technology associated with reduced polymerization shrinkage stress [39]. Manufacturer-reported information indicates that Filtek One Bulk Fill contains aromatic urethane dimethacrylate (AUDMA) and an addition-fragmentation monomer (AFM). AUDMA helps reduce the density of reactive groups and moderate shrinkage, whereas AFM is intended to promote stress relaxation during polymerization [42]. Nevertheless, both materials exhibited a similar deterioration pattern after prolonged hyperbaric exposure. These findings suggest that while material-related advantages—such as lower placement viscosity, higher filler loading, or shrinkage-stress control—may influence early marginal behavior, they do not appear sufficient to prevent long-term pressure-related degradation of the adhesive interface [14,23].

After 40 days of pressure exposure, both composites exhibited similarly elevated microleakage, indicating that neither viscosity modification nor shrinkage-reducing monomer strategies provided durable resistance against pressure-induced interface degradation [20,23].

These findings suggest that hyperbaric degradation should not be attributed to a single mechanism. Polymerization shrinkage stress may generate initial interfacial defects during curing, which are progressively exacerbated by long-term hyperbaric exposure through pressure-related fatigue. Similar pressure-related leakage patterns were reported by Mocquot et al. [23], Özyurt & Altınışık [20], and Shafigh et al. [24], collectively showing that short-term pressure has limited effects, but prolonged or stronger hyperbaric exposure causes greater leakage. These results therefore support a duration-dependent model of hyperbaric degradation.

From a clinical perspective, the present findings suggest that restorations in patients repeatedly exposed to hyperbaric environments may be at increased risk of marginal deterioration over time, particularly after prolonged treatment periods. Therefore, clinicians should be aware that patients undergoing repeated HBOT sessions may require closer follow-up of existing adhesive restorations. In such patients, careful restorative planning, meticulous placement to minimize void formation, and selection of materials with improved adaptation and resistance to interfacial degradation may be particularly important. These considerations may also be relevant for other individuals exposed to pressure fluctuations, such as divers and certain occupational groups.

A limitation of the present study is that the specimens were stored in distilled water, which cannot fully reproduce the dynamic oral environment. In vivo, the resin–dentin interface is exposed not only to water, but also to salivary enzymes, bacterial activity, pH fluctuations, thermal changes, and repeated mechanical loading, all of which may accelerate interfacial degradation. In addition, previous studies have shown that ageing strategy and storage medium can influence degradation behavior, and artificial saliva may produce different material responses compared with distilled water [43,44]. Furthermore, only one adhesive system was evaluated in the present study, limiting the generalizability of the conclusions. Therefore, these findings should be interpreted as specific to the adhesive protocol tested, rather than generalized to all adhesive systems; different adhesives may exhibit substantially different microleakage and bond durability after exposure to hyperbaric conditions. Because the present study focused primarily on microleakage outcomes, interfacial bond strength was not directly assessed. Since bond strength and microleakage are complementary but not interchangeable outcomes, the absence of bond strength testing limits the comprehensiveness of the present findings. Additional limitations include the lack of thermomechanical ageing, the use of a static rather than cyclic hyperbaric model, and the use of dye penetration alone to assess microleakage, without high-resolution imaging (e.g., micro-CT) of internal defects. Therefore, the present results should be interpreted as standardized in vitro findings, and direct clinical extrapolation should be made with caution. Future research should further investigate the clinical relevance of these findings by incorporating more realistic oral ageing conditions, including thermomechanical loading, artificial saliva, and cyclic pressure models. In addition, studies comparing different adhesive strategies, restorative materials, and interfacial bond strength outcomes would help clarify which material-related factors provide greater resistance to pressure-related degradation. Advanced imaging methods, such as micro-CT, may also improve understanding of internal defect formation and interfacial breakdown under hyperbaric conditions.

## 5. Conclusions

Prolonged exposure to elevated pressure significantly increased microleakage at the dentin–composite interface after 40 days, while shorter exposure periods did not produce noticeable changes. Both SonicFill and Filtek Bulk Fill composites exhibited similar susceptibility to pressure-related deterioration, despite differences in viscosity modification and resin chemistry. These results highlight that neither material offers long-term protection against hyperbaric stress, and emphasize the need for careful restorative planning in populations regularly exposed to pressure fluctuations.

## Figures and Tables

**Table 1:** Comparison of microleakage scores between composite groups (SonicFill vs. Filtek One Bulk Fill) by application type and time interval.

Application	Time	Leak Score	SonicFill n (%)	Filtek One Bulk Fill n (%)	SonicFill Median (IQR)	Filtek One Median (IQR)	*p*	r (Rank-Biserial)
Pressure (2.4 ATA)	Day 5	0	14 (87.5)	13 (81.2)	0 (0–0)	0 (0–0)	0.780	0.062
		1	2 (12.5)	3 (18.8)				
Pressure (2.4 ATA)	Day 20	0	12 (75.0)	11 (68.8)	0 (0–1)	0 (0–1)	0.724	0.078
		1	4 (25.0)	4 (25.0)				
		2	0 (0.0)	1 (6.2)				
Pressure (2.4 ATA)	Day 40	0	7 (43.8)	3 (18.8)	1 (0–2)	2 (1–2)	0.210	0.262
		1	3 (18.8)	4 (25.0)				
		2	6 (37.5)	9 (56.2)				
Distilled Water Control (1 Atm)	Day 5	0	16 (100.0)	15 (93.8)	0 (0–0)	0 (0–0)	0.780	0.062
		1	0 (0.0)	1 (6.2)				
Distilled Water Control (1 Atm)	Day 20	0	14 (87.5)	13 (81.2)	0 (0–0)	0 (0–0)	0.780	0.062
		1	2 (12.5)	3 (18.8)				
Distilled Water Control (1 Atm)	Day 40	0	13 (81.2)	12 (75.0)	0 (0–0)	0 (0–1)	0.780	0.062
		1	3 (18.8)	4 (25.0)				

Mann–Whitney U test. According to the Bonferroni correction, a *p* value < 0.0083 was considered statistically significant. r = rank-biserial correlation (effect size; |r| < 0.10 negligible, 0.10–0.30 small, 0.30–0.50 medium, >0.50 large). No significant differences were found between the composites at any time point or application condition.

**Table 2:** Comparison of microleakage scores between pressure (2.4 ATA) and distilled water control (1 Atm) conditions by composite and time interval.

Composite	Time	Leak Score	Pressure (2.4 ATA) n (%)	Distilled Water (1 Atm) n (%)	Pressure Median (IQR)	Distilled Median (IQR)	*p*	r (Rank-Biserial)
SonicFill	Day 5	0	14 (87.5)	16 (100.0)	0 (0–0)	0 (0–0)	0.564	0.125
		1	2 (12.5)	0 (0.0)				
SonicFill	Day 20	0	12 (75.0)	14 (87.5)	0 (0–1)	0 (0–0)	0.564	0.125
		1	4 (25.0)	2 (12.5)				
SonicFill	Day 40	0	7 (43.8)	13 (81.2)	1 (0–2)	0 (0–0)	0.032	0.445
		1	3 (18.8)	3 (18.8)				
		2	6 (37.5)	0 (0.0)				
Filtek One Bulk Fill	Day 5	0	13 (81.2)	15 (93.8)	0 (0–0)	0 (0–0)	0.564	0.125
		1	3 (18.8)	1 (6.2)				
Filtek One Bulk Fill	Day 20	0	11 (68.8)	13 (81.2)	0 (0–1)	0 (0–0)	0.515	0.137
		1	4 (25.0)	3 (18.8)				
		2	1 (6.2)	0 (0.0)				
Filtek One Bulk Fill	Day 40	0	3 (18.8)	12 (75.0)	2 (1–2)	0 (0–1)	<0.001	0.703
		1	4 (25.0)	4 (25.0)				
		2	9 (56.2)	0 (0.0)				

Mann–Whitney U test. According to the Bonferroni correction, a *p* value < 0.0083 was considered statistically significant. r = rank-biserial correlation (effect size; |r| < 0.10 negligible, 0.10–0.30 small, 0.30–0.50 medium, >0.50 large). No significant differences were found between the conditions at any time point or for any composite, except that Filtek One Bulk Fill at Day 40 showed a large effect (|r| = 0.703) and remained significant after correction.

**Table 3:** Evaluation of microleakage scores over time within each composite and application group.

Composite	Application	Leak Score	Day 5 n (%)	Day 20 n (%)	Day 40 n (%)	Med (IQR) D5	Med (IQR) D20	Med (IQR) D40	H	*p*	η^2^ (Effect Size)
SonicFill	Pressure (2.4 ATA)	0	14 (87.5)	12 (75.0)	7 (43.8)	0 (0–0)	0 (0–1)	1 (0–2)	9.744	0.008	0.172
		1	2 (12.5)	4 (25.0)	3 (18.8)						
		2	0 (0.0)	0 (0.0)	6 (37.5)						
Day 5 vs. Day 40: *p* = 0.009 ✓ | Day 5 vs. Day 20: *p* > 0.999, NS | Day 20 vs. Day 40: *p* = 0.061, NS											
SonicFill	Distilled Water Control (1 Atm)	0	16 (100.0)	14 (87.5)	13 (81.2)	0 (0–0)	0 (0–0)	0 (0–0)	3.060	0.216	0.024
		1	0 (0.0)	2 (12.5)	3 (18.8)						
Filtek One Bulk Fill	Pressure (2.4 ATA)	0	13 (81.2)	11 (68.8)	3 (18.8)	0 (0–0)	0 (0–1)	2 (1–2)	18.437	<0.001	0.365
		1	3 (18.8)	4 (25.0)	4 (25.0)						
		2	0 (0.0)	1 (6.2)	9 (56.2)						
Day 5 vs. Day 40: *p* < 0.0001 ✓ | Day 20 vs. Day 40: *p* = 0.002 ✓ | Day 5 vs. Day 20: *p* > 0.999, NS											
Filtek One Bulk Fill	Distilled Water Control (1 Atm)	0	15 (93.8)	13 (81.2)	12 (75.0)	0 (0–0)	0 (0–0)	0 (0–1)	2.056	0.358	0.001
		1	1 (6.2)	3 (18.8)	4 (25.0)						

KW = Kruskal–Wallis H statistic. η^2^ = eta-squared (effect size; η^2^ < 0.01 negligible, 0.01–0.06 small, 0.06–0.14 medium, >0.14 large). According to the Bonferroni correction, a *p* value < 0.0125 was considered statistically significant. Post hoc pairwise comparisons were performed using the Dunn–Bonferroni test to identify the source of the group differences. No significant time-dependent change was observed in either distilled water control group. ✓ indicates a statistically significant difference between the compared groups.

## Data Availability

The original contributions presented in this study are included in the article. Further inquiries can be directed to the corresponding author.
